# Nucleic acid protocols: Extraction and optimization

**DOI:** 10.1016/j.btre.2016.10.001

**Published:** 2016-10-05

**Authors:** Saeed El-Ashram, Ibrahim Al Nasr, Xun Suo

**Affiliations:** a State Key Laboratory for Agrobiotechnology, China Agricultural University, Beijing 100193, China; b National Animal Protozoa Laboratory & College of Veterinary Medicine, China Agricultural University, Beijing 100193, China; c Key Laboratory of Animal Epidemiology and Zoonosis of Ministry of Agriculture, Beijing 100193, China; d Faculty of Science, Kafr El-Sheikh University, Kafr El-Sheikh, Egypt; e College of Science and Arts in Unaizah, Qassim University, Unaizah, Saudi Arabia; f College of Applied Health Sciences in Ar Rass, Qassim University, Ar Rass 51921, Saudi Arabia

**Keywords:** Prokaryotic and eukaryotic sources, DNA, RNA, DNase, RNase

## Abstract

Yield and quality are fundamental features for any researchers during nucleic acid extraction. Here, we describe a simplified, semi-unified, effective, and toxic material free protocol for extracting DNA and RNA from different prokaryotic and eukaryotic sources exploiting the physical and chemical properties of nucleic acids. Furthermore, this protocol showed that DNA and RNA are under triple protection (i.e. EDTA, SDS and NaCl) during lysis step, and this environment is improper for RNase to have DNA liberated of RNA and even for DNase to degrade the DNA. Therefore, the complete removal of RNA under RNase influence is achieved when RNase is added after DNA extraction, which gives optimal quality with any protocols. Similarly, DNA contamination in an isolated RNA is degraded by DNase to obtain high-quality RNA. Our protocol is the protocol of choice in terms of simplicity, recovery time, environmental safety, amount, purity, PCR and RT-PCR applicability.

## Highlights

• A simplified, semi-unified, protocol for extracting DNA and RNA from different prokaryotic and eukaryotic sources.
• DNA and RNA are under triple protection (i.e. EDTA, SDS and NaCl) during lysis step.
• Adding DNase and RNase after DNA and RNA extraction respectively.

## Graphical abstract

**Figure:**
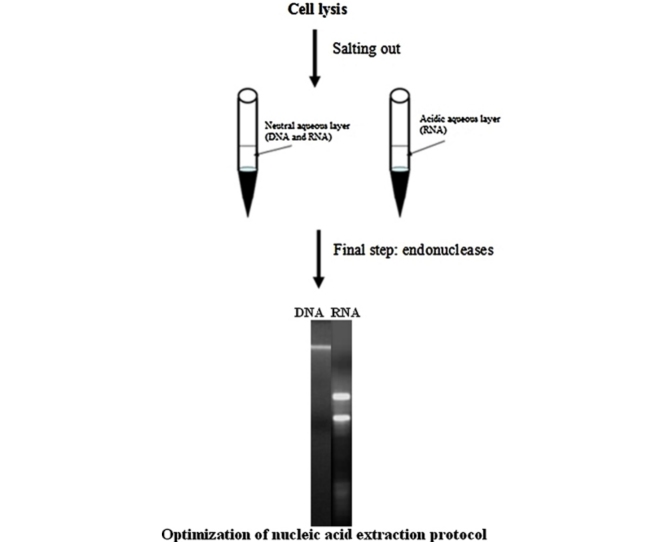

## 1 Introduction

Biomolecule extraction, such as deoxyribonucleic acid (DNA) and ribonucleic acid (RNA) from a variety of starting biological materials to be used in downstream applications and other analytical or preparative purposes, is the most important first step in the molecular biology. The widely employed nucleic acid isolation methods can be divided into organic extraction method (phenol/chloroform), inorganic extraction method (salting out) and solid phase extraction method (solid matrix); moreover, four indispensable steps are generally required for successful nucleic acid purification:

1. Cell lysis through disruption of the cellular membranes, cyst wall or egg wall
2. Dehydration and precipitation of the cellular proteins (protein denaturation)
3. Separation of cellular proteins and other cellular components out of the nucleic acid
4. Precipitation and dissolving the nucleic acid

The routinely practised cell lysis step can be divided into three types to cope with different tissues, thereby achieving optimum nucleic acid yield:

1. Grinding in liquid nitrogen (mortar and pestle), such as different animal and plant tissues
2. Glass-bead grinding, for example, oocysts (e.g. *Eimeria* spp.), metacercariae (e.g. *Fasciola* spp.) and nematodes’ eggs (e.g. eggs of *Haemonchus contortus*)
3. Repetitive pipetting, notable examples of it are animal cells and zoites of apicomplexan parasites, such as sporozoites, merozoites, tahyzoites and bradyzoites, and trypanosomal forms of *Trypanosoma* spp. and *Leishmania* spp., for example, trypomastigote, promastigote, amastigote and epimastigote.

In recent years, the development of molecular techniques has created a need for establishing simple and efficient novel methods of DNA and RNA extraction for PCR amplification and other related techniques. Carbohydrates, tannins, polyphenols and proteins in addition to hazardous organic solvents, such as phenol and chloroform are the major enemies of the embattled researchers. No existence for DNA or RNA extraction method that is suitable for all prokaryotic and eukaryotic organisms.

Furthermore, there is an urgent need to address the insufficiency of reasonable environment for RNase to have DNA free of RNA and even for DNase to degrade the DNA.

## 2 Materials and methods

### 2.1 Reagents

Proteinase K, 100% Ethanol, 70% Ethanol, Double distilled (DD) water, Ethylene diamine tetra acetic acid (EDTA), RNase, DNase, Pyrex beads, Agarose, Deoxyribonucleic acid (DNA) Marker, 2 × EasyPfu PCR SuperMix, 10% Sodium dodecyl sulfate (SDS), Glacial acetic acid (CH_3_COOH), Hydrochloric acid (HCl) and Sodium hydroxide (NaOH).

### 2.2 Equipments

Mortar, Pestle, PCR machine, Microscope, Refrigerated Benchtop centrifuge (MIKRO200R, Germany), Weighing scale, Pipettes (20, 100, and 1000 μl), 15 and 50 ml falcon tubes, 50 ml centrifuge tubes and Disposable Polypropylene micro-centrifuge tubes

### 2.3 Reagent setup

Tris buffer, Tris-EDTA (TE), DEPC-treated water, Saturated salt solution (NaCl), Neutral saturated salt solution, Acidic saturated salt solution and Lysis buffer:1X STE buffer (50 mM NaCl, 50 mM Tris-HCl and 100 mM EDTA; PH 8.0)

### 2.4 Procedure

#### 2.4.1 Grinding in liquid nitrogen (Mortar and pestle)

##### 2.4.1.1 DNA extraction protocol

Hepatic DNA extraction from mouse can be divided into six steps. These are:

###### 2.4.1.1.1 Homogenization

1 g of the liver was taken and cut into pieces then ground using a porcelain mortar and pestle in 3 ml of lysis buffer containing 900 μl of 10% SDS. The emulsion was transferred to micro-centrifuge tubes and 100 μg proteinase K was added per ml of emulsion solution, and incubated for 1 h at 50 °C.

###### 2.4.1.1.2 Phase separation

**Fig. 1:**
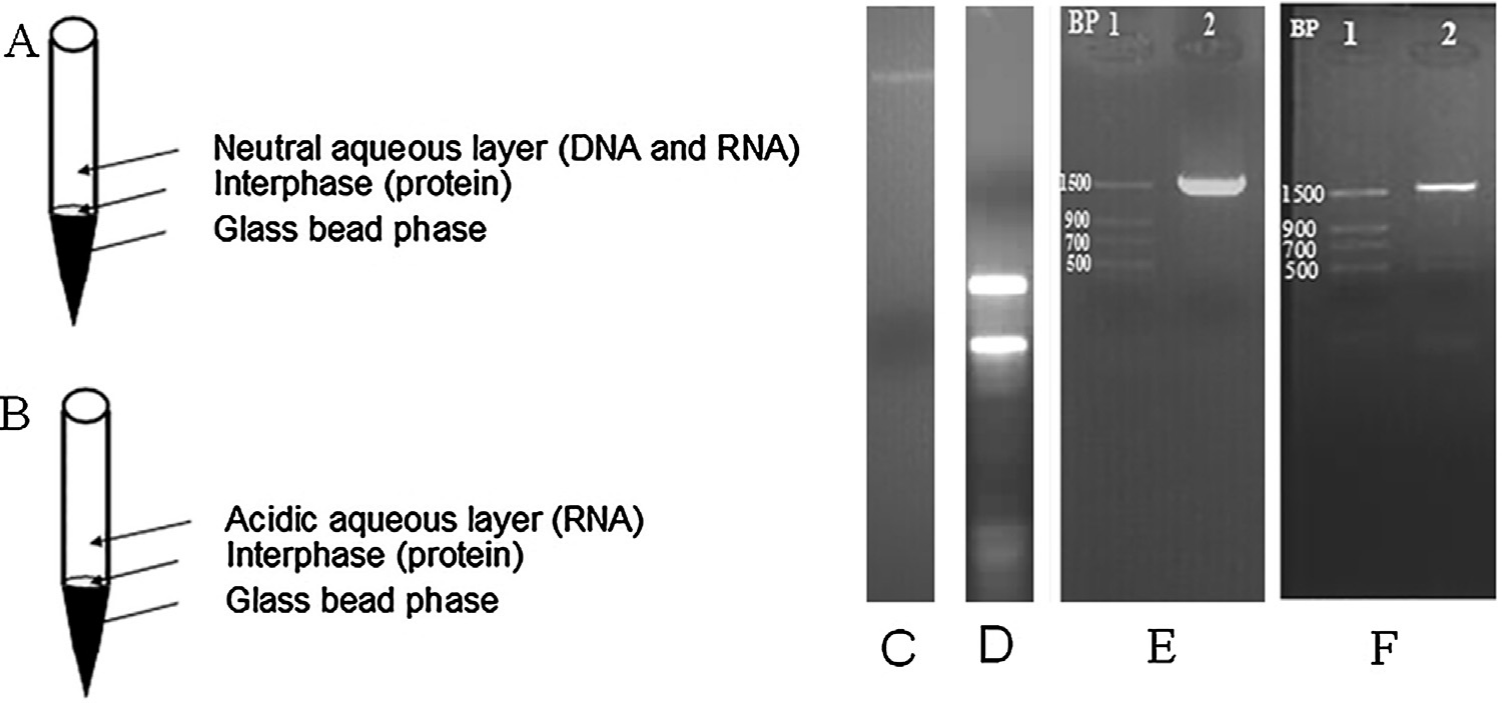
Nucleic acid extraction and downstream application. A. Neutral salting out (DNA extraction). B. Acidic salting out (RNA extraction). C. GoldView™ Nucleic Acid Stained 1.5% Agarose gel demonstrating the integrity of total DNA extracted from *Eimeria tenella*. D. GoldView™ Nucleic Acid Stained 1.5% Agarose gel of total RNA of *E. tenella*. E. Standard PCR amplification of MICII of *E. tenella.* F. GoldView™ Nucleic Acid Stained 1.5% Agarose gel of EtMIC-2 RT-PCR amplified product of *E. tenella* using Finnzymes phusion™ High-Fidelity DNA Polymerase.

350 μl of neutral saturated salt solution (NaCl) per ml was added to the previous emulsion, the micro-centrifuge tube was capped and shaken gently by hand for 15 s, and then incubated at room temperature for 10 min. The micro-centrifuge tube was centrifuged at 590 × *g* for 15 min at room temperature with DNA remaining exclusively in the aqueous phase (see Fig. 1A for illustration).

###### 2.4.1.1.3 DNA precipitation

The resulting aqueous phase was transferred into another micro-centrifuge tube, and mixed with two volumes of room temperature absolute ethyl alcohol. Then the micro-centrifuge tube was inverted several times for 10 s.

###### 2.4.1.1.4 DNA wash

The supernatant was removed; the DNA pellet was washed once with 75% ethanol, and the DNA was precipitated out of the solution by centrifugation at 9500 × *g* for 5 min.

###### 2.4.1.1.5 DNA dissolving

The DNA pellet was allowed to dry for 5 min, and dissolved in DD water. Then the DNA was quantified and aliquoted to be stored at −20 °C.

###### 2.4.1.1.6 Removal of RNA from DNA preparation

50 μg per ml RNase was added and the mixture was incubated for 1 h at 37 °C.

• Critical step: *The treatment of DNA with RNase should be done in Tris buffer at the end of the extraction protocol. Salting out step can be repeated as before according to the protocol to obtain DNA with highest quality. The DNA can be precipitated and washed with 70% ethanol, and then the pellet can be dissolved in Tris-EDTA (TE) for DNA protection from degradation by metal dependent nucleases during storage.*

##### 2.4.1.2 RNA extraction protocol

Hepatic RNA extraction method from mouse can be listed as follows:

###### 2.4.1.2.1 Homogenization

1 g of the liver was taken and cut into pieces then ground using a porcelain mortar and pestle in 3 ml of lysis buffer containing 900 μl of 10% SDS. The emulsion was transferred to micro-centrifuge tubes.

###### 2.4.1.2.2 Phase separation

350 μl of acidic saturated salt solution (NaCl) was added into each tube of the previous emulsion mixture, and the micro-centrifuge tube was capped and gently shaken by hand for 15 s and then incubated at room temperature for 10 min. The micro-centrifuge tube was centrifuged at 590 × *g* for 15 min at room temperature with RNA remaining exclusively in the aqueous phase (see Fig. 1B for elucidation).

###### 2.4.1.2.3 RNA precipitation

The resulting aqueous phase was transferred into micro-centrifuge tubes and precipitated by mixing the aqueous phase with two volumes of cold absolute ethyl alcohol. Then the micro-centrifuge tube was inverted several times for 15 s.

###### 2.4.1.2.4 RNA wash

The supernatant was removed; and the RNA pellet was washed once with cold 75% ethanol, and the RNA was precipitated out of the solution by centrifugation at 9500 × *g* for 5 min.

###### 2.4.1.2.5 RNA dissolving

The RNA pellet was allowed to dry for 5 min and dissolved in DEPC-treated water. Then the RNA was quantified, and aliquoted to be stored at −80 °C.

###### 2.4.1.2.6 DNA removal

DNase I (RNase-free) kit was employed to remove any contaminating DNA from the sample as per manufacturer’s instructions.

• Critical step: *The treatment of RNA with DNase should be done in Tris buffer at the end of the extraction protocol. Salting out step can be repeated as before according to the protocol to obtain RNA with highest quality. The RNA can be precipitated and washed with ethanol, and then the pellet can be dissolved in DEPC-treated water or Tris-EDTA (TE) for DNA protection from degradation by metal-dependent nucleases during storage.*

#### 2.4.2 Repetitive pipetting: prokaryotes, for example *E. coli*

##### 2.4.2.1 DNA extraction protocol

Bacterial DNA extraction can be listed as follows:

###### 2.4.2.1.1 Homogenization

1 ml microbial culture was transferred into micro-centrifuge tubes and pelleted by centrifugation at 380 × *g* for 5 min at room temperature. Then the supernatant was discarded, and the pellet was resuspended with repetitive pipetting in 1 ml lysis buffer containing 100 μl of 10% SDS and 100 μg proteinase K. The mixture was incubated for 1 h at 50 °C.

###### 2.4.2.1.2 Phase separation, DNA precipitation, DNA wash, DNA dissolving and. RNA removal

They were conducted as previously mentioned in Section 2.4.1.1.

##### 2.4.2.2 RNA extraction protocol

Bacterial RNA extraction can be divided into six sections:

###### 2.4.2.2.1 Homogenization

1 ml microbial culture in microcentrifuge tube was pelleted by centrifugation at 380 × *g* for 5 min at 37 °C, and the supernatant was discarded. The pellet was resuspended with repetitive pipetting in 1 ml lysis buffer containing 100 μl of 10% SDS.

###### 2.4.2.2.2 Phase separation, RNA precipitation, RNA wash, RNA dissolving and DNA removal

They were performed as we previously mentioned in Section 2.4.1.2.

#### 2.4.3 Glass-bead grinding

##### 2.4.3.1 DNA extraction protocol

Eimerian DNA extraction from oocysts can be divided into six steps. These are:

###### 2.4.3.1.1 Homogenization

5 × 10^6^ sporulating/sporulated oocysts, 0.5 g of Pyrex beads and up to 3 ml of lysis buffer containing 900 μl of 10% SDS and 300 μg proteinase K were added in a 15 ml disposable polypropylene tube. The mixture was incubated for 1 h at 50 °C.

###### 2.4.3.1.2 Phase separation

1 ml of neutral saturated salt solution was added to the previous mixture, and the 15 ml disposable polypropylene tube was capped and gently shaken by hand for 15 s and then incubated at room temperature for 10 min. The 15 ml disposable polypropylene tube was centrifuged at 590 × *g* for 15 min at room temperature.

###### 2.4.3.1.3 DNA precipitation, DNA wash, DNA dissolving and removing contaminating RNA from DNA

These steps were then performed as previously reported in Section 2.4.1.1.

##### 2.4.3.2 RNA extraction protocol

Eimerian RNA extraction can be divided into six sections:

###### 2.4.3.2.1 Homogenization

5 × 10^6^ sporulating/sporulated oocysts, 0.5 g of Pyrex beads and up to 3 ml of lysis buffer containing 900 μl of 10% SDS were added in a 15 ml disposable polypropylene tube, and the mixture was incubated for 1 h at 42 °C.

###### 2.4.3.2.2 Phase separation

1 ml of acidic saturated salt solution was added to the previous mixture, and the 15 ml disposable polypropylene tube was capped and gently shaken by hand for 15 s and then incubated at room temperature for 10 min. The 15 ml disposable polypropylene tube was centrifuged at 590 × *g* for 15 min at room temperature.

###### 2.4.3.2.3 RNA precipitation, RNA wash, RNA dissolving and DNA removal

They were conducted as we previously reported in Section 2.4.1.2.

## 3 Results and discussion

### 3.1 Eukaryotes, such as

#### 3.1.1 *Eimeria* spp.

##### 3.1.1.1 DNA isolation

The average DNA purity A_260_/A_280_ ratio was 1.87 ± 0.025 of three repeated samples, which indicates low amounts of contaminants in the samples. The quantity of DNA extracted from 6 × 10^6^ sporulated eimerian oocysts was 3.5985 ± 0.27 mg with integrity validated by 1.5% Agarose gel electrophoresis (Fig. 1C).

##### 3.1.1.2 RNA isolation

The average RNA purity A_260_/A_280_ ratio was 1.42 ± 0.035 with yield average of 191.7 ± 1.7 μg/6 × 10^6^ unsporulated eimerian oocysts. As can be seen from Fig. 1D, the integrity of isolated total RNA was confirmed by 1.5% Agarose gel electrophoresis.

##### 3.1.1.3 Assessing DNA and RNA for downstream applications

The ability to amplify a specific target from extracted DNA and RNA was proved using a pair of precise primers; MIC2-UP TATGGCTCGAGCGTTGTCGCTG and MIC2-D GTCAGGATGACTGTTGAGTGTC that were designed from the published *Eimeria tenella* microneme 2 (MIC2) mRNA sequence (ACCESSION FJ807654) (Fig. 1E). The primers were synthesized by AuGCT Biotechnology Synthesis Lab, Beijing, China. Fig. 1F presents RT-PCR amplification of MICII of *E. tenella*.

#### 3.1.2 BALB/c mice

##### 3.1.2.1 DNA isolation

**Fig. 2:**
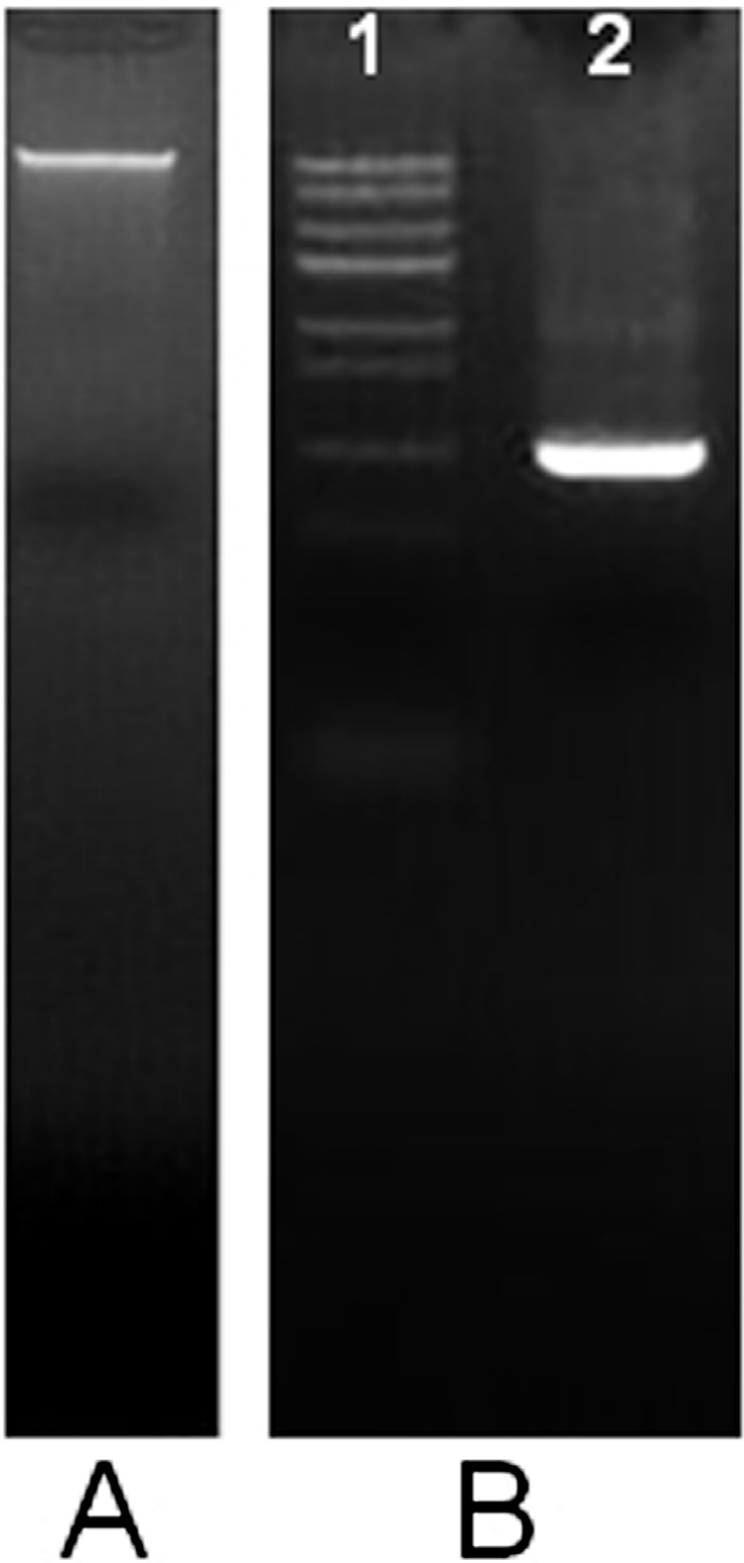
GoldView™ Nucleic Acid Stained 1.5% Agarose gel demonstrating. A. The integrity of total DNA extracted from the liver of the BALB/c mice. B. GAPDH amplified product of mouse genome. Lane 1: Trans DNA Marker III, Lane 2 and 3: GAPDH amplified product from the genomic DNA of mouse genome.

The average A_260_/A_280_ ratio and yield of the total DNA extracted were 1.83 ± 0.025 and 20 ± 0.45 μg/g of liver tissue, respectively. The integrity of isolated total DNA was confirmed by 1.5% Agarose gel electrophoresis as shown in Fig. 2A.

##### 3.1.2.2 Assessing DNA for downstream applications

The ability to amplify a specific target, such as 16S Ribosomal DNA from extracted DNA was proved using a pair of precise primers; forward GAPDH Primer,

5′-CAAGGTCATCCATGACAACTTTG-3′ and the reverse GAPDH Primer,

5′-GTCCACCACCCTGTTGCTGTAG-3′ that were provided with RevertAid™ First Strand cDNA Synthesis Kit to test the control sample (Fig. 2B).

### 3.2 Prokaryotic examples; *E. coli*

#### 3.2.1 DNA isolation

**Fig. 3:**
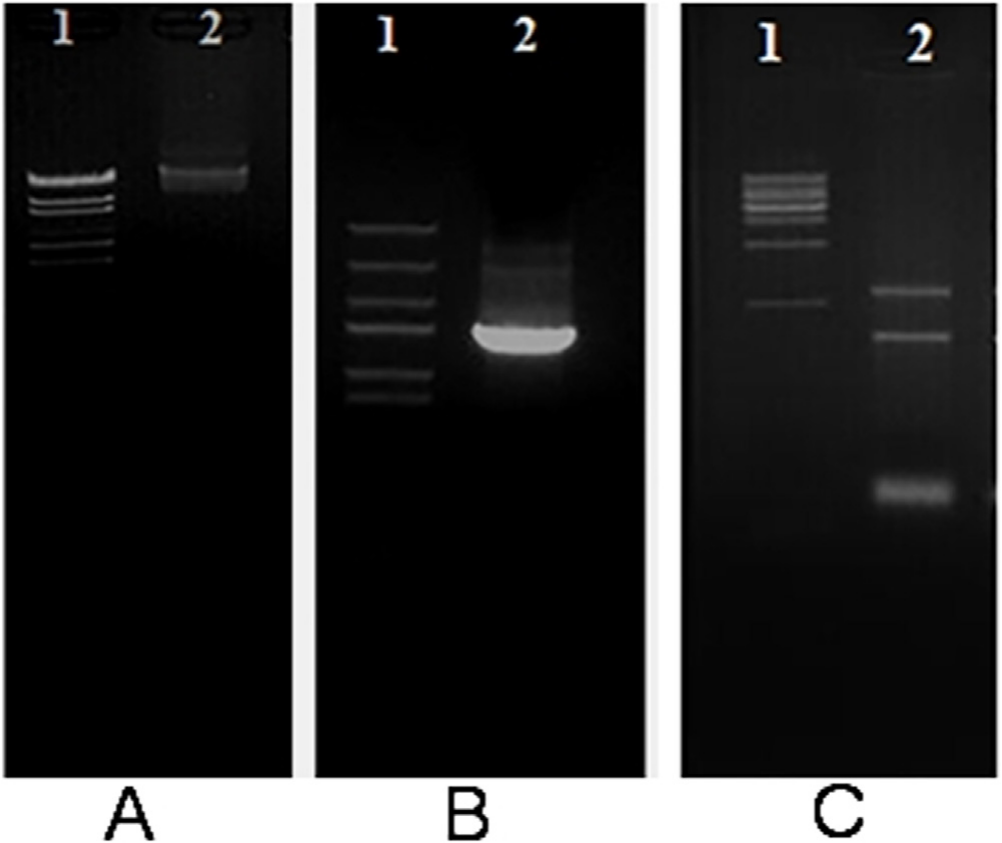
GoldView™ Nucleic Acid Stained 1.5% Agarose gel illustrating. A. The integrity of total DNA extracted from the *Escherichia coli* bacterial cultures. Lane 1 λ-EcoT14 I digest DNA Marker, Lane 2 Genomic DNA of the *Escherichia coli.* B. 16S ribosomal RNA sequence amplified product of *E. coli* genome. Lane 1: Trans DNA Marker III, Lane 2 16S ribosomal RNA amplified product from the genomic DNA of *E. coli* genome. C. Total RNA of *E. coli*. Lane 1 Trans DNA Marker 1Kb DNA ladder, Lane 2 total RNA of *E. coli.*

The average DNA purity ratio A_260_/A_280_ was 1.87 ± 0.065 with DNA yield average of 48 ± 2.24 μg/1 ml (1 × 10^6^ cells) of *Escherichia coli* bacterial cultures that were grown overnight in Luria Broth (LB) at 37 °C. The integrity of isolated total DNA was confirmed by 1.5% Agarose gel electrophoresis as presented in Fig. 3A.

#### 3.2.2 Assessing DNA for downstream applications

The ability to amplify a specific target, such as the *E.coli* 16S ribosomal RNA sequence from extracted DNA was proved using a pair of precise primers that were designed from the published *E.coli* 16S ribosomal RNA sequence (ACCESSION NO J01859/K02555/M24828/M24833/M24834/M24835/M24836/M24837/M24911/M24996) (Fig. 3B). The primers were synthesized by AuGCT Biotechnology Synthesis Lab, Beijing, China.

#### 3.2.3 RNA isolation

The average purity of RNA samples was A_260_/A_280_ ratio was 1.99 ± 0.01 and the quantity of RNA extracted from 1 × 10^6^ *E. coli* was 22 ± 1.45 μg with integrity confirmed by 1.5% Agarose gel electrophoresis as can be seen from Fig. 3C.

### 3.3 Further assessment of RNA purity and integrity using trichostrongylid adult worm

**Fig. 4:**
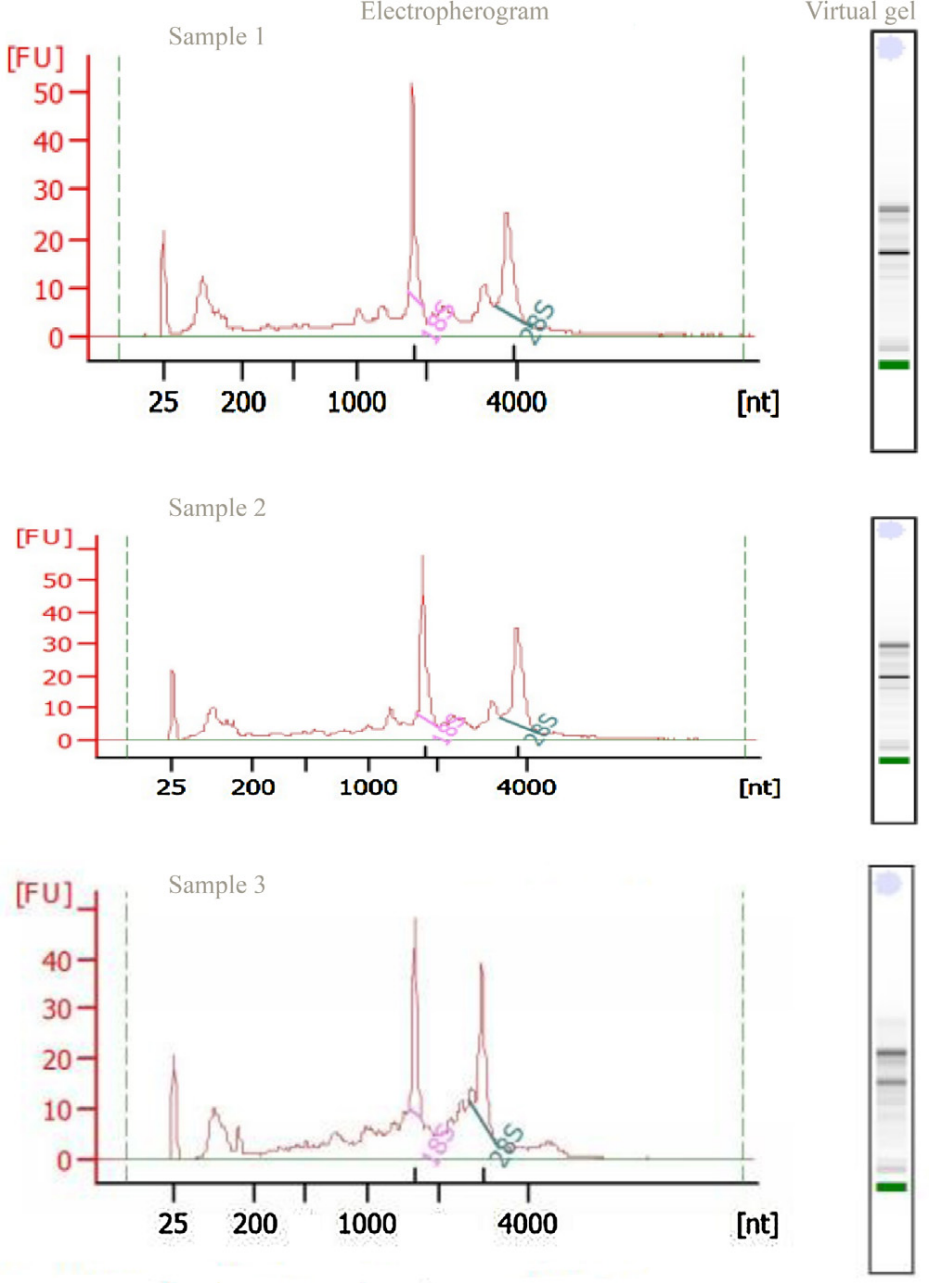
Electropherogram and gel image output (virtual gel) from Agilent Bioanalyzer's *Haemonchus contortus* total RNA nano assay. The x-axis represents fluorescence unit (FU) and y-axis represents time (seconds).

Total RNA from the barber’s pole worm, *Haemonchus contortus* was isolated employing salting out (acidic condition) followed by DNase digestion. For contaminant detection, the A_260_/A_280_ and A_260_/A_280_ values (i.e. for detection of protein contaminants and residual chemical contamination, such as EDTA and SDS) were 2 ± 0.02 and 2.076 ± 0.024, respectively [1], [2], [3]. Additionally, Agilent Bioanalyzer 2100 system (Agilent Technologies, CA, USA) was used in conjunction with the traditional 1.5% agarose gels for RNA integrity assessment (Fig. 4, Table 1).

These results present a simplified, semi-unified, effective, and toxic material free protocol for extracting DNA and RNA from different prokaryotic and eukaryotic sources exploiting the physical properties of the negatively charged molecules; DNA and RNA. The positively ions of saturated salt solution neutralize the negatively charged phosphate groups of the DNA and RNA backbone. Furthermore, in neutral saturated salt conditions, DNA will remain in the aqueous layer. However, RNA will partition into the aqueous layer by carrying out acidic saturated salt solution extraction.

Yield and quality are the ultimate goal for any researchers during DNA extraction procedure. Doubtless, the quality increases by getting RNA free of DNA contamination. Previous published studies failed to resolve this issue [4], [5], [6], [7], [8], [9], [10], [11], [12], and (http://www.natureprotocols.com/2009/05/27/a_protocol_for_high_molecular.php, http://www.nwfsc.noaa.gov/publications/techmemos/tm14/dnaisol.html, http://csm.jmu.edu/biology/courses/bio480_580/mblab/genomic2.htm, www.promega.com).

**Fig. 5:**
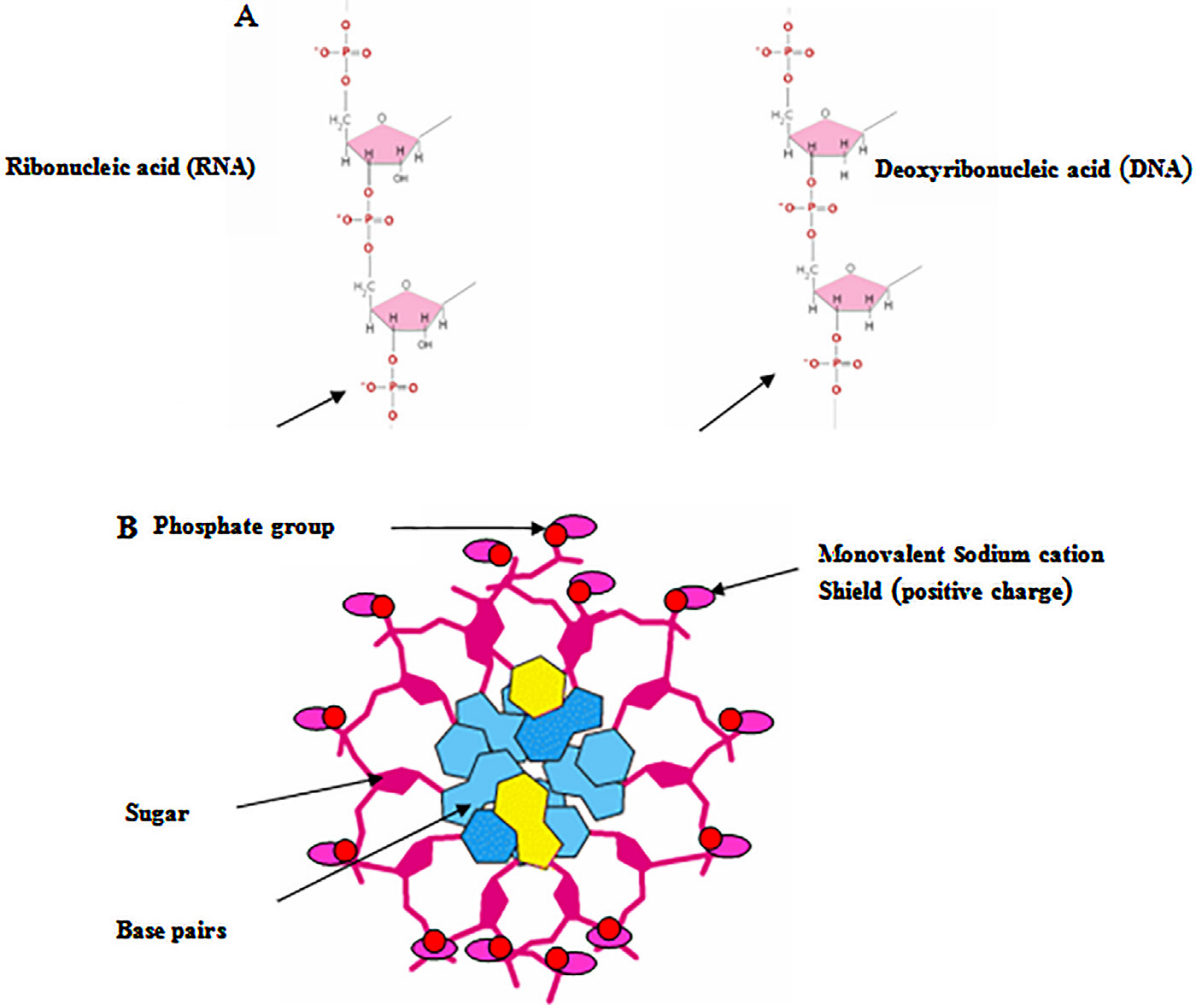
Diagrams illustrate the negatively charged phosphate (PO_4_^3−^) of DNA and RNA molecules and the positively charged monovalent sodium cation shield around the helix axis. A. Negatively charged phosphate group in the ribonucleic acid and deoxyribonucleic acid backbone. B. Axial view of DNA with sodium cation shield modified from Berg et al., 2002.

The most common protocols used the chelating agent, ethylenediaminetetraacetic acid (EDTA), sodium dodecyl sulfate (SDS) as a detergent, and sodium chloride as a stabilizer in the lysis buffer. The high affinity of EDTA to divalent cations, such as Ca^2+^, Mn^2+^ and Mg^2+^, which act as cofactors for nucleases could inhibit the degradation of DNA and RNA by DNases and RNases respectively [13], [14], [15]. SDS is an anionic detergent for cell and nucleus lysis to release ribonucleic and deoxyribonucleic acids. The nucleases; ribonuclease (RNase) and deoxyribonuclease (DNase) activities were inhibited by SDS [16], [17]. The electrostatic repulsion between the two negatively charged helix strands destabilizing the helix was counteracted by positively charged sodium chloride [18]. The shielding effect of monovalent sodium cations leads to DNA and RNA stabilization through neutralization of the negative charge on the sugar phosphate backbone as is demonstrated in Fig. 5 [19].

Elevated salt concentration, SDS and EDTA were used to inhibit nuclease activity during extraction of DNA from tissues or organisms with high nuclease activity [20]. The use of sodium chloride in the lysis buffer decreases the susceptibility of DNA and RNA to be attacked by the action of nucleases possibly due to steric hindrance. Additionally, salting out denatures proteins and leaves nucleic acids intact. This is the most potent way of expeditiously inactivating nucleases.

**Fig. 6:**
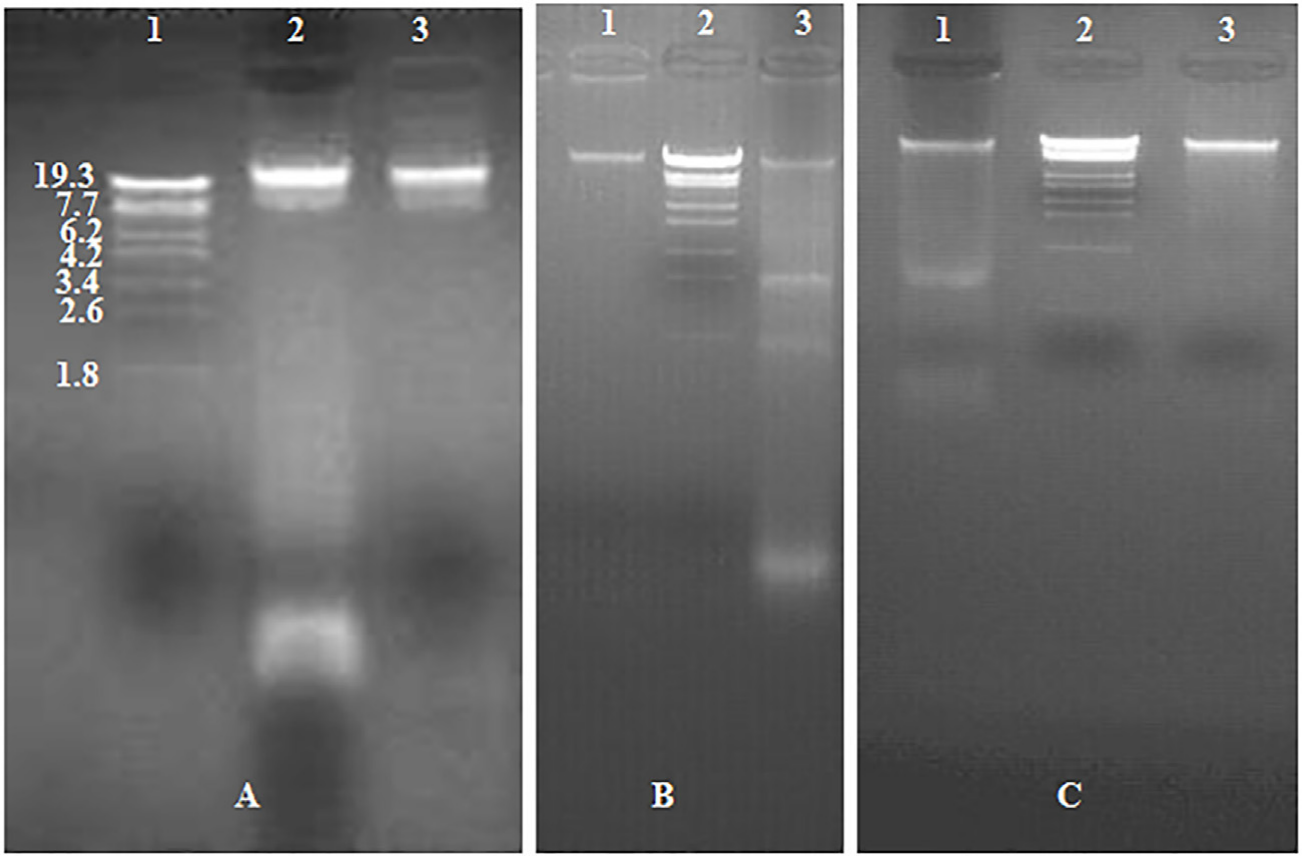
GoldView™ Nucleic Acid Stained 1.5% Agarose gel of total DNA isolated from different prokaryotic and eukaryotic sources using our current protocol for DNA and RNA isolation. A. Lane 1: λ-EcoT14 I digest DNA Marker, Lane 1 and 2: Genomic DNA of *E. coli* using RNase at the cell lysis step Lane 2 and during the dissolving step Lane 3. B. Lane 2: λ-EcoT14 I digest DNA Marker, Lane 1 and 2: Genomic DNA of *E. tenella* BJ strain using RNase at the cell lysis step Lane 3 and during the dissolving step Lane 1. C. Lane 2: λ-EcoT14 I digest DNA Marker. Lane1: Genomic DNA of mouse using RNase at the cell lysis step and Lane 3 and during the dissolving step.

We pointed out that DNA and RNA are under triple protection (i.e. EDTA, SDS and NaCl) and this environment is unsuitable for RNase to get DNA free of RNA and even for DNase to degrade DNA. Our conclusion is supported by results from treatments of different prokaryotic and eukaryotic sources as illustrated in Figs. 6A–C. The complete removal of RNA under the effect of RNase is achieved when RNase is eventually added (i.e. in Tris buffer without EDTA), which gives optimal quality with any DNA extraction protocols.

The polar phosphate groups of DNA and RNA can electrostatically interact with the polar environment allowing them to be easily dissolved in Tris buffer; therefore, the treatment of DNA and RNA with RNase and DNase respectively should be done in Tris buffer at the end of the extraction protocol as a standard measure. Salting out step can be repeated as before according to the protocol to obtain DNA with the highest quality without major changes in the nucleic acid yield. The DNA can be precipitated and washed with ethanol, and then the pellet can be dissolved in Tris-EDTA (TE) for DNA and RNA protection from degradation by divalent-metal-dependent nucleases during storage.

## 4 Conclusion

A simplified, semi-unified, effective, and toxic material free protocol for extracting DNA and RNA utilizing the physicochemical properties of nucleic acids has been described. Moreover, the unsuitable environment for endonucleases, such as RNase and DNase to have DNA liberated of RNA and even for DNase to degrade the DNA respectively has been addressed, and an appropriate alternative protocol has been presented that could be the top-first in the field of molecular biology.

## Conflict of interest

The authors declare that there are not conflicts of interest.

## Figures and Tables

**Table 1:** Test results of Agilant 2100.

Parameters	Average ± standard deviation (SD)
RNA concentration^a^	164.7 ± 13.6 μg/g
RNA integrity number (RIN)^a^	7.27 ± 0.32

a Each value is a mean of three separate samples.

## References

[1] Maniatis T., Fritsch E.F., Sambrook J. (1982). Molecular Cloning A Laboratory Manual, Cold Spring Harbor Laboratory.

[2] Wilfinger W. William, Mackey Karol, Chomczynski Piotr (2016). Effect of pH and ionic strength on the spectrophotometric assessment of nucleic acid purity. Biotechniques.

[3] Tataurov A.V., You Y., Owczarzy R. (2008). Predicting ultraviolet spectrum of single stranded and double stranded deoxyribonucleic acids. Biophys. Chem..

[4] Wallach M., Cully D.F., Haas L.O.C., Trager W., Cross G.A.M. (1984). Histidine rich protein genes and their transcripts in *Plasmodium falciparum* and *p. lophurae*. Mol. Biochem. Parasitol..

[5] Wallach M.M., Mencher D., Yarus S., Pillemer G., Halabi A., Pugatsch T. (1989). *Eimeria maxima*: identification of gametocyte protein antigens. Exp. Parasitol..

[6] Braun R., Shirley M.W.P. (1995). Special techniques of molecular biology. Biotechnology Guidelines on Techniques in Coccidiosis Research. Cost 89/820.

[7] Penney M., Wilkinson C., Wallace M., Javerzat J., Ferrell K., Seeger M., Dubiel W., McKay S., Allshire R., Gordon C. (1998). The pad1+ gene encodes a subunit of the 26S proteasome in fission yeast. J. Biol. Chem..

[8] Garner I. (2000). The Nucleic Acid Protocols Handbook.

[9] Sharma A., Singh J.A. (2005). Nonenzymatic method to isolate genomic DNA from bacteria and actinomycete. Anal. Biochem..

[10] Di Pinto A., Forte V.T., Guastadisegni M.C., Martino C., Schena F.P., Tantillo G. (2007). A comparison of DNA extraction methods for food analysis. Food Control.

[11] Ogunkanmi L.A., Oboh B., Onifade B., Ogunjobi A.A., Taiwo I.A., Ogundipe O.T. (2008). An improved method of extracting genomic DNA from preserved tissues of *Capsicum annuum* for PCR amplification. EurAsian J. BioSci..

[12] Kahánková J., Španová A., Pantůček R., Horák D., Doškař J., Rittich B. (2009). Extraction of PCR-ready DNA from Staphylococcus aureus bacteriophages using carboxyl functionalized magnetic nonporous microspheres. J. Chromatogr. B.

[13] Rosenberg N.L. (1987). ATP as an alternative inhibitor of bacterial its effect on native chromatin compaction and endogenous nucleases and its effect on native chromatin compaction. Mol. Cell Biochem..

[14] Adams R.L.P., Knowler J.T., Leader D.P. (1992). The Biochemistry of the Nucleic Acids.

[15] Van Wert S.L., Saunders J.A. (1992). Reduction of nuclease activity released from germinating pollen under conditions used for pollen electrotransformation. Plant Sci..

[16] Williams J.A., Yeggy J.P., Field C.C., Markovetz A.J. (1980). Role of nucleases in the isolation of plasmid deoxyribonucleic acid from *Pseudomonas cepacia* 4G9. J. Bacteriol..

[17] Farrell R.E. (2010). RNA Methodologies: A Laboratory Guide for Isolation and Characterization.

[18] Dale J.W., Von Schantz M. (2002). From Genes to Genomes: Concepts and Applications of DNA Technology.

[19] Berg J.M., Tymoczko J.L., Stryer L. (2002). Biochemistry.

[20] Karp A., Isaac P.G., Ingram D.S. (1998). Molecular Tools for Screening Biodiversity: Plants and Animals.

